# Genomic Biomarkers for Personalized Medicine: Development and Validation in Clinical Studies

**DOI:** 10.1155/2013/865980

**Published:** 2013-04-17

**Authors:** Shigeyuki Matsui

**Affiliations:** Department of Data Science, The Institute of Statistical Mathematics, 10-3 Midori-cho, Tachikawa, Tokyo 190-8562, Japan

## Abstract

The establishment of high-throughput technologies has brought substantial advances to our understanding of the biology of many diseases at the molecular level and increasing expectations on the development of innovative molecularly targeted treatments and molecular biomarkers or diagnostic tests in the context of clinical studies. In this review article, we position the two critical statistical analyses of high-dimensional genomic data, gene screening and prediction, in the framework of development and validation of genomic biomarkers or signatures, through taking into consideration the possible different strategies for developing genomic signatures. A wide variety of biomarker-based clinical trial designs to assess clinical utility of a biomarker or a new treatment with a companion biomarker are also discussed.

## 1. Introduction

Advances in biotechnology and genomics have gradually uncovered the biology of many diseases and the heterogeneity among diseases with the same diagnosis at the molecular level. Deeper understanding of disease biology can facilitate the development of new treatments, while deeper understanding of the disease heterogeneity can facilitate the development of effective biomarkers or diagnostic tests for selecting appropriate treatments for individual patients. In particular, the recent establishment of high-throughput molecular assay technologies, such as single-nucleotide polymorphism (SNP) arrays, gene expression microarrays, and protein arrays, has allowed discovery of potential new biomarkers and development of composite genomic signatures for personalized medicine. 

The establishment of high-throughput technologies, at the same time, has stimulated the application of data-driven analytical approaches for high-dimensional genomic data from high-throughput assays. In the development of genomic signatures, the data-driven approaches are typically supervised in the sense that the information of a particular clinical variable, such as response to a particular treatment and survival outcomes after treatments, is utilized in analyzing genomic data. Specifically, two important statistical approaches are identified: (1) screening of relevant genetic features for subsequent studies and (2) building of genomic classifiers or predictors for a clinical variable. The high-dimensionality of the genomic data, however, has posed special challenges to extracting a small fraction of relevant signals in the presence of a large amount of noise variables. A large amount of biostatistical or bioinformatical methods have been proposed in the context of the development of genomic biomarkers [1–5]. 

For clinical application of a developed biomarker toward personalized medicine, the validity and clinical utility of the biomarker need to be evaluated in the context of clinical studies. Randomized clinical trials are a gold standard for evaluating the clinical utility of the biomarker itself or a new treatment associated with the aid of the biomarker. Recently, various biomarker-based designs of randomized clinical trials have been proposed and applied. 

This paper is organized as follows. After identifying a class of biomarkers essential for personalized medicine and providing important criteria for biomarker validation in Section 2, we provide a review of the critical statistical tasks, gene screening and prediction analysis, for the development of genomics biomarkers in Section 3. Biomarker-based designs for randomized clinical trials to evaluate clinical utility are outlined in Section 4. Finally, concluding remarks will appear in Section 5. 

## 2. Biomarkers for Personalized Medicine and Their Validation Criteria

### 2.1. Predictive and Prognostic Biomarkers

Two types of biomarkers are particularly important for personalized medicine: *predictive* and *prognostic* biomarkers. Predictive biomarkers are pretreatment or baseline measurements that provide information about which patients are likely or unlikely to benefit from a specific treatment. A predictive biomarker is often designated for the use of a particular new treatment, as a companion predictive biomarker in the development of the new treatment. As a typical example in oncology, a biomarker that captures overexpression of the growth factor protein Her-2, which transmits growth signals to breast cancer cells, can be a predictive biomarker for treating breast cancer patients using trastuzumab (Herceptin) which blocks the effects of Her-2. Prognostic biomarkers are pretreatment measurements that provide information about the long-term outcome of untreated patients or those receiving the standard treatment. Prognostic biomarkers reflect the baseline risk and may not necessarily indicate responsiveness to a particular treatment like predictive biomarkers, but they can suggest some treatment for patients undergoing a standard treatment. Patients who are predicted to have a poor prognosis would require a more aggressive treatment, while patients who are predicted to have a sufficiently good prognosis would not require additional treatments.

### 2.2. Criteria for Biomarker Validation

The criteria for validating a biomarker should depend on the intended use of the biomarker. Three different types of validation have been proposed for predictive and prognostic biomarkers: analytical validation, clinical validation, and clinical utility [6, 7]. 

Analytical validation refers to establishment of robustness and reproducibility of the assay and accuracy of measurement, such as sensitivity and specificity, relative to a gold standard assay if one is available [8]. 

Clinical validity refers to establishment of the ability of the biomarker in predicting prognosis or treatment effects in individual patients. For a prognostic biomarker, correlation between biomarker status and a clinical endpoint (such as survival time) may indicate clinical validity. For reliable clinical validation of a predictive biomarker for a clinical endpoint, a randomized clinical trial would be required to estimate treatment effects (of a new treatment relative to a control treatment) unbiasedly and to assess whether the treatment effects vary depending on the status of the biomarker, that is, a treatment-by-biomarker interaction. 

Lastly, clinical utility requires that the biomarker is actionable in clinical practice and the use of the biomarker results in improved outcome of patients and leads to patient benefit [7]. Therefore, one critical element in establishing clinical utility is to evaluate the improved patient outcomes associated with the use of the developed prognostic biomarker, through comparing with those based on a standard of care without the biomarker. In the codevelopment of a new treatment and a companion predictive biomarker, treatment effects associated with the use of the developed predictive biomarker will be evaluated. Evaluation of clinical utility through randomized clinical trials will be outlined in Section 4.

## 3. Statistical Approaches for Developing Genomic Signatures

This section provides a review of statistical approaches used for the development of genomic signatures. We mainly suppose the development of gene expression signatures and the use of DNA microarrays as high-throughput assays. Other types of genomic analyses, such as single-nucleotide polymorphism genotyping, copy number proofing, and proteomic profiling data, can be used similarly for developing genomic signatures.

### 3.1. Gene Screening

Most high-throughput technologies to date have been used primarily as a research tool, and therefore, some conversion from a high-throughput platform into a platform that is more applicable to clinical practice would be needed. For example, in measuring gene expressions, quantitative polymerase chain reaction (PCR) assays are such a clinical platform. Many quantitative PCR assays are known to be highly specific, sensitive, and robust, compared with the high-throughput microarrays, but can measure only small numbers of genes at one time in a single sample, unlike the microarray platform (e.g., [9]). This may necessitate limiting the number of candidate genes, when converting from the microarray platform into the clinical platform. 

The standard strategy for developing genomic signatures is to base them on established clinical platforms, such as PCR platforms. In order to incorporate the possible limitation in the number of genes that can be investigated in the PCR platform, the most popular approach is to screen out a small number of relevant genes from a pool of a large number of gene candidates in the earlier microarray study and, after conversion to a clinical platform, to build a predictor based on the selected genes using the data measured in the clinical platform. This strategy was taken in the development of the Oncotype Dx signature for recurrence risk classification of breast cancer [10] and the AlloMap signature for rejection surveillance after cardiac transplantation [11]. 

#### 3.1.1. Multiple Testing

The most popular statistical approach for gene screening is to apply multiple testing methodologies that perform separate statistical tests for each gene to test the null hypothesis of no association with the clinical variable. For example, in comparing normalized gene expression levels (log signals from oligonucleotide arrays or log ratios from two-color spotted cDNA arrays) between two phenotypic classes, for gene *g*, the two-sample *t*-statistic is calculated, $Y_{g}=({\hat{\mu }}_{g}^{\left(1\right)}-{\hat{\mu }}_{g}^{\left(2\right)})/{\hat{\sigma }}_{g}$, aside from the sample-size constant, *τ*_*n*_^2^ = *n*/(*n*_1_ *n*_2_), so that *T*_*j*_ = *Y*_*j*_/*τ*_*n*_. Here, ${\hat{\mu }}_{g}^{\left(1\right)}$ and ${\hat{\mu }}_{g}^{\left(2\right)}$ are the mean expression levels for classes 1 and 2, respectively, and ${\hat{\sigma }}_{g}$ is a pooled estimate of the within-class standard deviation for gene *g*  (*g* = 1,…, *G*) using the data from the two classes with sample sizes of *n*_1_ and *n*_2_, so that the total number of samples is *n* = *n*_1_ + *n*_2_. The results of the *G* tests can be summarized as a contingency table as shown in Table 1. Note that whether the null or alternative status is true is unknown for each gene. Because the conduction of many statistical tests sharply increases the number of false positives, some control of false positives is mandatory (e.g., [12]). The false discovery rate (FDR) [13] is commonly employed as a criterion for controlling false positives in the multiple testing of high-dimensional genomic data [12, 14]. This is defined as the expected proportion of false positives among the genes declared significant, FDR = *E*(*V*/*R*). When *R* = 0, the proportion *V*/*R* is defined to be 0, since no null hypothesis is rejected.

Control for false positives, however, will yield a serious lack of power in multiple testing. The efficacy of multiple testing can be improved by borrowing the strength across genes by assuming exchangeability across comparable genes and modeling the underlying structure for the data set across genes. A multitude of frequentist, empirical Bayes, and full Bayes methods have been developed [1–5, 15]. A simple model [16] is the following hierarchical mixture model for the distribution of *Y*_*j*_:

(1) $$\begin{array}{c} f\left(y\right)=\pi f_{0}\left(y\right)+\left(1-\pi \right)f_{1}\left(y\right), \end{array}$$

where *f*_0_ and *f*_1_ are the density functions of *Y* for null and non-null genes, respectively, and null or non-null genes occur with prior probabilities of *π* or 1 − *π*, respectively. We can assume the theoretical null *N*(0, *τ*_*n*_^2^) for *f*_0_. For the non-null component, *f*_1_, we assume the following hierarchical structure:

(2) $$\begin{array}{c} Y_{g}\;|\;\delta_{g}\sim N\left(\delta_{g},\tau_{n}^{2}\right),\;\;\delta_{g}\sim h_{1}. \end{array}$$

In the first level, given a gene-specific mean *δ*_*g*_ = (*μ*_*g*_^(1)^ − *μ*_*g*_^(2)^)/*σ*_*g*_, *Y*_*g*_ follows a normal distribution. In the second level, the gene-specific *δ*_*g*_ follows a distribution *h*_1_.

However, one of the most effective approaches for controlling true positives or overall power, such as *E*(*S*/*G*_1_), is the determination of the number of biological replicates, *n*. In sample size estimation, accurate assessment of the strength of the “signal” contained in the data set, represented by the parameters, such as the proportion of non-null genes 1 − *π* and the effect size distribution for non-null genes *h*_1_ is crucial because these parameters can largely impact the sample size estimates [16]. 

#### 3.1.2. Ranking and Selection

The ranking and selection methodologies, that are used to rank genes based on the magnitude of association or effect sizes and select a given number of top ranking genes with the largest effect sizes, can be a more practical approach to incorporate the limitation in the number of genes that can be investigated in the subsequent studies based on the clinical platform [17–20]. Simple univariate statistics, such as fold change for two-class comparison, ${\hat{\mu }}_{g}^{\left(1\right)}-{\hat{\mu }}_{g}^{\left(2\right)}$, can be used for gene ranking [21]. Recently, more accurate gene ranking methods that borrow the strength across genes via hierarchical mixtures modeling such as (1) and (2) have been proposed [19, 20]. 

#### 3.1.3. Remarks

Advantages of the multiple testing and gene ranking approaches relate to the ease in interpreting the output from these analyses based on the marginal association between single genes and the clinical variable. Importantly, these approaches are usually complemented by incorporation of external information from biological considerations (such as annotation regarding gene function categories and partial information regarding genetic pathways) and knowledge from previous similar screening studies. Typically, biologists and statisticians cooperatively narrow the list down to a subset of genes of limited size that can be investigated in subsequent studies based on the clinical platform. 

These univariate approaches are often criticized because of the lack of consideration of plausible correlations among genes in gene screening. Thus, there is no guarantee that a set of selected genes is optimal in terms of prediction accuracy. One rationale for employing the univariate approaches could be given by selection of genes strongly associated with the clinical outcome that are essential for improving predictive accuracy significantly over that of existing clinical biomarkers or diagnostics [22]. 

When candidate genes that may pass through to the clinical platform are identified, possibly incorporating biological considerations and knowledge from previous correlative studies, it is worthwhile to assess whether classification or prediction using a candidate subset of selected gene is in fact promising or worth using in progression to subsequent phases for developing molecular diagnostics. A wide variety of standard prediction models (e.g., [23]) are applicable for relatively small numbers of the variable (gene) after the preliminary gene screening. For binary classification, a misclassification rate can be estimated based on shrinkage estimates (posterior means) of the standardized effect sizes *δ*_*g*_ for any set of selected genes in the framework of hierarchical modeling [22, 24]. Unlike the standard framework of prediction analysis (see Section 3.2), an independent test sample would not be needed to assess the accuracy of classification.

### 3.2. Prediction Analysis

Recent advance in biotechnology has allowed the development of new high-throughput platforms for clinical application. As an early endeavor in this direction, in the development of the MammaPrint signature for recurrence risk classification of breast cancer, a custom microarray was developed to allow clinical application of the prediction system based on gene expressions of 70 genes developed in an experimental microarray platform [25, 26]. More recently, in the development of the Tissue of Origin Test for identifying tumor tissue of origin [27], a new microarray platform was developed to measure gene expressions of a pool of almost 5,000 genes. This new platform can work with formalin-fixed, paraffin-embedded tissue specimens that may contain degraded RNA, the clinical standard to tissue fixation and processing for the purpose of diagnostic histology and long-time storage. The advent of such new high-throughput assays may allow developing genomic signatures in the same (high-throughput) platform, throughout all of the processes of the development and analytical/clinical validation of genomic signatures. From the perspective of statistical analysis, we would now be free of the limitation in the number of genes for building predictors as is required in the traditional developmental strategies based on standard clinical platforms.

#### 3.2.1. Development of Predictor

When building a predictor using high-dimensional genomic data, where the number of variables (genes) is much greater than the number of samples, traditional regression modeling is ineffective. Traditional approaches ensure that all of the variables included would fail in estimation or result in overfitted models with poor prediction ability. Some sort of dimension reduction or regularization is needed. A large amount of prediction techniques under high-dimension have been developed (e.g., [1–5, 23]). 

Filtering methods described in Section 3.1.1 for incorporating the limitation in the number of genes in the strategy with standard clinical platforms can be effective for accurate prediction because a large proportion of genes would be noisy and useless for prediction. Recent studies found that the performance of univariate filtering methods, based on marginal association between each gene and the clinical variable, was comparable to that of multivariate methods for microarray datasets with small sample sizes [28, 29].

With regard to prediction model building, some studies on class prediction reported that simple methods that ignore correlations between genes, such as diagonal linear discriminant analysis and *k*-nearest neighbors, performed well in terms of prediction accuracy compared with more complex methods such as aggregated classification trees for microarray datasets with small-to-moderate sample sizes [30–32]. 

#### 3.2.2. Clinical Validation of Predictors

Unbiased estimation of the predictive accuracy is particularly important when the number of candidate variables (genes) available for use in the predictor is much greater than the number of samples available for analysis. For class prediction problems, the proportion of correct classification, sensitivity, and specificity are common measures of predictive accuracy.

In high-dimensional situations, one must focus clearly on the objective of accurate prediction and not confuse this objective with that of achieving biological insight or ensuring that all variables included are essential, or that the model is “correct” [33, 34]. For example, a prognostic genomic signature might contain a gene that is only representative of a group of highly correlated prognostic genes. With slightly different data, a different gene from that group might be selected. Therefore, the signature will be rather unstable with different interpretations, while prediction performance may not be affected much [35]. In other words, there might exist many “solutions” of predictor with comparable predictive accuracy under high dimension. For example, several prognostic signatures developed for breast cancer had little overlap of the component genes, but showed comparable prediction accuracy [36]. Reproducibly of the gene list reported among similar correlative studies, which can be critical in elucidating the underlying biological mechanisms, can mislead in the assessment of genomic signatures for predictive medicine. 

For assessment of predictive accuracy, a completely specified genomic signature is needed. Complete specification of the signature includes not only the list of component genes, but also the mathematical form used to combine genomic data for the genes used in the signature, weights for the relative importance of the genes, and cut-off values when making classification [34]. 

Assessment of predictive accuracy includes internal and external validation. The internal validation is to assess predictive accuracy for the study population from which the predictor was built, typically using validation techniques such as split-sample or cross-validation. On the other hand, the external validation is performed using an independent set of samples, possibly from a more relevant population for clinical application of the predictor. For example, in the development of the Oncotype Dx, the predictive accuracy of the developed predictor (the recurrence score based on 21 genes to classify three recurrence risk groups) was assessed in a PCR-based platform for an independent cohort from another clinical trial [10].

For the assessment of internal validity in high dimensions, resampling techniques such as cross-validation and bootstrap are useful, particularly when the sample size is small [37, 38]. When using these techniques, it is critical that all aspects of model building including gene selection are reperformed for each round in resampling [39, 40]. When selection of genes and prediction models are optimized based on cross-validated predictive accuracy, the optimization process should be included in the cross-validation procedure or an independent validation set is needed to have an unbiased estimate of the predictive accuracy [41, 42]. If the cross-validated predictive accuracy measures without incorporating the optimization process are relatively insensitive to selection of the tuning parameters used in the optimization, this bias may not be large. Confidence intervals for cross-validated prediction error can also be calculated [43]. It is also important to establish that the predictive accuracy is statistically higher than that expected when there is no relationship between genomic data and the clinical variable. A permutation procedure is proposed to assess the statistical significance of cross-validated predictive accuracy [44].

When the model building process is complex and not easily specified in an algorithmic manner, an independent validation set would be needed [3]. Some authors provided a formula for determining sample sizes for the training and validation sets [45].

## 4. Biomarker-Based Clinical Trial Designs for Assessing Clinical Utility 

This section outlines various biomarker-based designs for randomized clinical trials to evaluate clinical utility. We can identify at least two types of such biomarker-based designs based on their primary objectives. One is to establish clinical utility for the developed biomarker or genomic signature itself, through comparing to the standard of care without using the biomarker. The biomarker-strategy designs have such an objective. Another type is to establish clinical utility for a new treatment under development with the aid of a predictive biomarker, or for the pair of a new treatment and its companion predictive biomarker. The enrichment designs and the randomize-all designs have such an objective. 

### 4.1. Biomarker Strategy Designs

**Figure 1:**
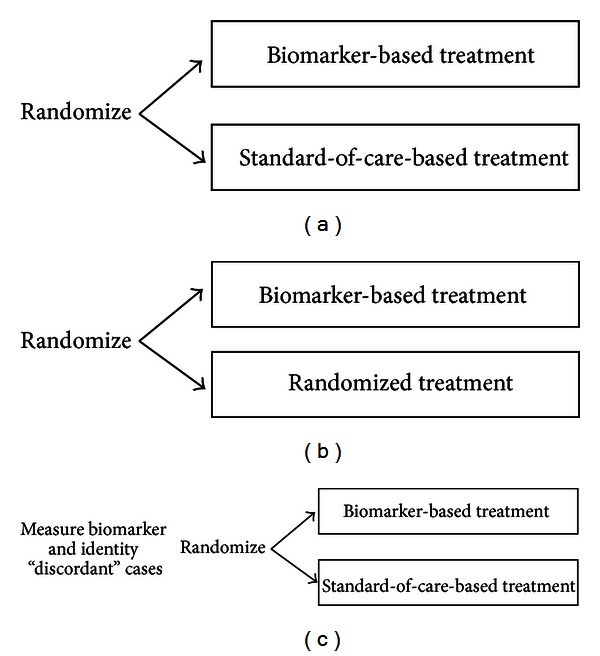
Biomarker strategy designs.

With a biomarker strategy design, patients are randomized either to a strategy of using the biomarker in determining their treatment or to a strategy of not using the biomarker in determining treatment. The primary objective is, thus, to compare two strategies with and without use of the biomarker in determining treatment. An example is a randomized trial for recurrent ovarian cancer that compares the strategy of determining treatment based on tumor chemosensitivity (predictive) assays with a strategy of using physician's choice of chemotherapy based on standard practice [46] (see Figure 1(a)). Another example is a randomized trial for non-small-cell lung cancer that compares a strategy of using a standard treatment (cisplatin + docetaxel) exclusively with a biomarker-based strategy in which patients diagnosed to be resistant to the standard treatment based on the biomarker are treated with an experimental treatment (gemcitabine + docetaxel) and the rest are treated with the standard treatment [47] (see Figure 1(b)). In these designs, the biomarker is evaluated only for the patients assigned to the biomarker-based strategy arm.

For the latter type of design with an experimental treatment, the biomarker-based arm can perform better if the experimental treatment is efficacious, regardless of whether the biomarker is predictive or not. Some authors proposed a modification in which patients in the nonbiomarker-based arm undergo a second randomization to receive one of the same two treatments being used in the biomarker-based arm, that is, the control and experimental treatments [48]. By measuring the biomarker status in all of the patients, the modified design would allow clinical validation of the biomarker as a predictive biomarker, through comparing treatment effects across the biomarker-based subsets of patients. 

The strategy-based designs fundamentally include patients treated with the same treatment in both the biomarker-based and the non-biomarker-based arms, resulting in a large overlap in the number of patients receiving the same treatment within the two strategies being compared. Thus, a very large number of patients are required to be randomized to detect a diluted, small overall difference in the endpoint between the two arms. One modification is to randomize the two strategies to only the patients for whom the two treatments guided by the two strategies differ (see Figure 1(c)). This modification requires measurement of the biomarker in all of the patients before randomization. The modified design is generally much more efficient than the original biomarker strategy design. The modified design was employed in a randomized clinical trial, called the MINDACT study. In this trial, a biomarker-based strategy based on the MammaPrint prognostic signature was compared to that based on standard clinical prognostic factors for determining whether to utilize chemotherapy in women with node-negative estrogen receptor-positive breast cancer, in which discordant cases between the two strategies were subject to randomization [49]. 

### 4.2. Enrichment Designs

**Figure 2:**
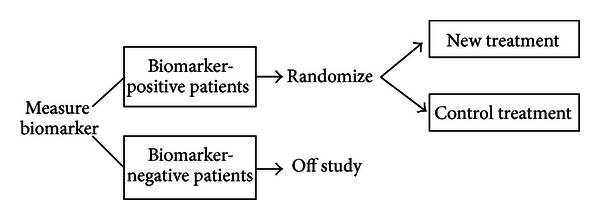
Enrichment design.

An enrichment or targeted design is based on a predictive biomarker and compares a new treatment and a control treatment only in biomarker-“positive” patients who are expected to be responsive to the new treatment based on the biomarker (see Figure 2). Thus, the enrichment design assesses treatment efficacy in the biomarker-based subset of patients, and not in the entire patient population. In this design, patients need to be screened for evaluating the biomarker status. 

The efficiency of the enrichment design relative to the standard approach of randomizing all patients without using the biomarker at all depends on the prevalence of biomarker-positive patients and on the effectiveness of the new treatment in biomarker-negative patients [50, 51]. In particular, when fewer than half of the patients are biomarker-positive and the new treatment is relatively ineffective in biomarker-negative patients, the enrichment design can be conducted with much smaller numbers of randomized patients. The enrichment design was employed in the development of trastuzumab; metastatic breast cancer patients whose tumors expressed Her2 in an immunohistochemistry test were eligible for randomization [52]. 

The enrichment design is appropriate for contexts where there is compelling biological evidence for believing that biomarker-negative patients will not benefit from the new treatment and that including them would raise ethical concerns [7]. In addition, before initiating the trial, the biomarker used for enrichment must be analytically validated with established assay accuracy, reproducibility, and robustness. 

When the biological basis is not compelling and/or assay accuracy is incomplete, assessment of clinical validity of the biomarker as a predictive biomarker would be needed. As the enrichment design does not allow it because of the absence of comparison of the new treatment with the control in biomarker-negative patients, the following designs with randomization of both biomarker-positive and -negative patients; that is, randomize-all designs are an alternative choice for such situations. 

### 4.3. Randomize-All Designs

#### 4.3.1. Designs with a Single, Completely Specified Biomarker

**Figure 3:**
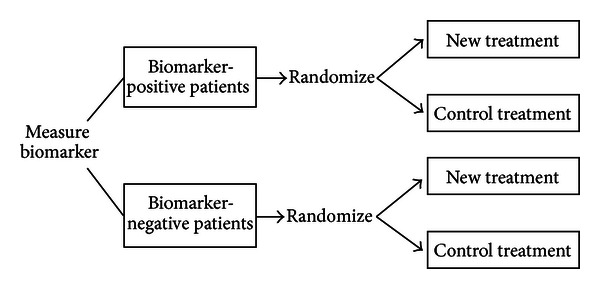
Randomize-all design with prestratification based on the biomarker.

When there is no compelling biological data or data from early trials for a completely specified biomarker candidate regarding its capability in predicting treatment effects, it is generally reasonable to include all patients as eligible for randomization, that is, randomize-all designs, as done in the traditional paradigm, but to entail prospective subset analysis based on the predictive biomarker [7, 34, 48, 53–55]. Randomization can be either unstratified or stratified on the basis of the predictive biomarker. Stratification may ensure that all randomly assigned patients have biomarker status observed (see Figure 3).

These designs can demonstrate the efficacy of the treatment for either the overall population or a biomarker-based subset of patients, through inspecting the predictive capability of the biomarker candidate based on the observed trial data. These designs are, thus, composed of an adaptive analysis. Various designs with a single biomarker candidate have been proposed, including by-biomarker fixed-sequence designs, fallback designs, and treatment-by-biomarker-interaction designs.

When the biological basis for a candidate biomarker is strong, so that one is unlikely to expect the treatment to be effective in the biomarker-negative patients, unless it is effective in the biomarker-positive patients, the following by-biomarker fixed-sequence design would be suggested [7, 34]. At the first stage, one compares the treatment versus control in biomarker-positive patients at a significance level of 5%. If this test is significant, we proceed to the second stage, or otherwise stop the analysis. At the second stage, we compare the treatment versus control in biomarker-negative patients at a significance level of 5%. This sequential approach controls the overall type I error at 5%. 

When there is limited confidence in the predictive biomarker, it is generally reasonable to assess treatment efficacy for the overall patient population and prepare the subset analysis as a fallback option. Specifically, at the first stage, the experimental treatment is compared with the control treatment overall at a reduced significance level *α*_1_, such as 0.03. If this test is significant, then treatment efficacy is demonstrated for the overall population. Otherwise, at the second stage, the experimental treatment is compared with the control in the biomarker-positive patients at a reduced significance level *α*_2_, such as 0.02 [54, 56]. The significance level *α*_2_ can be specified by taking into account the correlation between the first test for the overall population and the second test for the subset of biomarker-positive patients [54, 57]. Another design when there is limited confidence in the predictive biomarker is to decide whether to compare treatments overall or within the biomarker-based subsets based on a preliminary test of interaction of treatment and biomarker [7, 34, 48]. 

#### 4.3.2. More Complex Designs with Biomarker Development and Validation

When the biology of the target of a new treatment is not well understood because of the complexity of disease biology, it is quite common that a complete specified predictive biomarker is not available before initiating the definitive phase III trial. One approach for such situations is to design and analyze the randomized phase III trial in such a way that both developing a predictive biomarker and testing treatment efficacy based on the developed biomarker are possible and conducted validly. 

Jiang et al. [58] developed the adaptive threshold design for settings where a single predictive biomarker candidate is available but no threshold of positivity for the biomarker is predefined. The basic idea is, for a set of candidate threshold values (*b*_1_, …, *b*_*K*_), to search for an optimal threshold value through maximizing a log likelihood ratio of treatment effect for the patients with biomarker value  ≥  *b*_*k*_ over possible threshold values (*k* = 1,…, *K*). The maximum log likelihood ratio at the optimal threshold value is used as the test statistic. Its null distribution is approximated by repeating the whole analysis after randomly permuting treatment levels several thousand times. 

Another adaptive design, called adaptive signature design, is to develop a predictor or signature using a set of covariates *x*, possibly high-dimensional genomic data [59, 60]. As the second stage of the fallback designs, the full set of patients in the clinical trial is partitioned into a training set and a validation set. A prespecified algorithmic analysis plan is applied to the training set to generate a predictor. This is a function of *x* and predicts whether a given patient with a particular value of *x* will be responsive or not responsive to the new treatment. The predictor is used to make a prediction for each patient in the validation set. Then, the treatment efficacy is tested in the subset of patients who are predicted to be “responsive” to the treatment in the validation set. This modified second-stage analysis of the fallback designs can be based on split-sample [59] or cross-validation [60]. 

Recently, Matsui et al. [61] developed another framework designed to estimate treatment effects quantitatively as a function of a continuous cross-validated predictive score for the entire patient population, rather than qualitatively classifying patients as being in or not in a responsive subset. Average absolute treatment effects for the entire population or a responsive subset of patients can be estimated based on the estimated treatment-effects function and tested using a permutation method. In this framework, patient-level survival curves can be developed to predict survival distributions of future individual patients as a function of the cross-validated predictive score and a cross-validated prognostic score.

## 5. Concluding Remarks

Recent advances in biotechnology and genomics have stimulated further research of biostatistical and bioinformatics methodologies for the development and validation of new genomic biomarkers or diagnostic tests that are useful for selecting the right treatments for the right patients. The established heterogeneity of disease based on genomic biomarkers then warrants the development of new paradigms of design and analysis of clinical trials for assessing the validity and clinical utility of new treatments and the companion biomarkers toward reliable personalized or predictive medicine.

## Figures and Tables

**Table 1:** Possible outcomes from *G* hypothesis tests.

	Significant	Not significant	Total
Null true	V	*G*_0_ − *V*	*G*_0_
Alternative true	S	*G*_1_ − *S*	*G*_1_

Total	R	*G* − *R*	*G*

## References

[1] Speed T (2003). *Statistical Analysis of Gene Expression Microarray Data*.

[2] McLachlan GJ, Do K-A, Ambroise C (2004). *Analyzing Microarray Gene Expression Data*.

[3] Simon RM, Korn EL, McShane LM, Radmacher MD, Wright GW, Zhao Y (2004). *Design and Analysis of DNA Microarray Investigations*.

[4] Dehmer M, Emmert-Streib F (2013). *Statistical Diagnostics For Cancer: Analyzing High-Dimensional Data*.

[5] Matsui S, Noma H, Crowley JJ, Hoering A (2012). Analysis of DNA microarrays. *Handbook of Statistics in Clinical Oncology*.

[6] Hunter DJ, Khoury MJ, Drazen JM (2008). Letting the genome out of the bottle—will we get our wish?. *The New England Journal of Medicine*.

[7] Simon R (2010). Clinical trial designs for evaluating the medical utility of prognostic and predictive biomarkers in oncology. *Personalized Medicine*.

[8] Chau CH, Rixe O, McLeod H, Figg WD (2008). Validation of analytic methods for biomarkers used in drug development. *Clinical Cancer Research*.

[9] Erickson HS (2012). Measuring molecular biomarkers in epidemiologic studies: laboratory techniques and biospecimen considerations. *Statistics in Medicine*.

[10] Paik S, Shak S, Tang G (2004). A multigene assay to predict recurrence of tamoxifen-treated, node-negative breast cancer. *The New England Journal of Medicine*.

[11] Pham MX, Teuteberg JJ, Kfoury AG (2010). Gene-expression profiling for rejection surveillance after cardiac transplantation. *The New England Journal of Medicine*.

[12] Dupuy A, Simon RM (2007). Critical review of published microarray studies for cancer outcome and guidelines on statistical analysis and reporting. *Journal of the National Cancer Institute*.

[13] Benjamini Y, Hochberg Y (1995). Controlling the false discovery rate: a practical and powerful approach to multiple testing. *Journal of the Royal Statistical Society B*.

[14] Dudoit S, Popper-Shaffer J, Boldrick JC (2003). Multiple hypothesis testing in microarray experiments. *Statistical Science*.

[15] Dey DK, Ghosh S, Mallick BK (2011). *Bayesian Modeling in Bioinformatics*.

[16] Matsui S, Noma H (2011). Estimating effect sizes of differentially expressed genes for power and sample size assessments in microarray experiments. *Biometrics*.

[17] Pepe MS, Longton G, Anderson GL, Schummer M (2003). Selecting differentially expressed genes from microarray experiments. *Biometrics*.

[18] Matsui S, Zeng S, Yamanaka T, Shaughnessy J (2008). Sample size calculations based on ranking and selection in microarray experiments. *Biometrics*.

[19] Noma H, Matsui S, Omori T, Sato T (2010). Bayesian ranking and selection methods using hierarchical mixture models in microarray studies. *Biostatistics*.

[20] Noma H, Matsui S (2012). Empirical Bayes ranking and selection methods via semiparametric hierarchical mixture models in microarray studies. *Statistics in Medicine*.

[21] Shi L, Reid LH, Jones WD (2006). The MicroArray Quality Control (MAQC) project shows inter- and intraplatform reproducibility of gene expression measurements. *Nature Biotechnology*.

[22] Matsui S, Noma H (2011). Estimation and selection in high-dimensional genomic studies for developing molecular diagnostics. *Biostatistics*.

[23] Trevor H, Robert T, Jerome F (2009). *The Elements of Statistical Learning: Data Mining, Inference and Prediction*.

[24] Efron B (2009). Empirical bayes estimates for large-scale prediction problems. *Journal of the American Statistical Association*.

[25] van de Vijver MJ, He YD, van ’t Veer LJ (2002). A gene-expression signature as a predictor of survival in breast cancer. *The New England Journal of Medicine*.

[26] van’t Veer LJ, Dai H, van de Vijver MJ (2002). Gene expression profiling predicts clinical outcome of breast cancer. *Nature*.

[27] Monzon FA, Lyons-Weiler M, Buturovic LJ (2009). Multicenter validation of a 1,550-gene expression profile for identification of tumor tissue of origin. *Journal of Clinical Oncology*.

[28] Lai C, Reinders MJT, van’t Veer LJ, Wessels LFA (2006). A comparison of univariate and multivariate gene selection techniques for classification of cancer datasets. *BMC Bioinformatics*.

[29] Lecocke M, Hess K (2006). An empirical study of univariate and genetic algorithm-based feature selection in binary classification with microarray data. *Cancer Informatics*.

[30] Ben-Dor A, Bruhn L, Friedman N, Nachman I, Schummer M, Yakhini Z (2000). Tissue classification with gene expression profiles. *Journal of Computational Biology*.

[31] Dudoit S, Fridlyand J, Speed TP (2002). Comparison of discrimination methods for the classification of tumors using gene expression data. *Journal of the American Statistical Association*.

[32] Shi L, Campbell G, Jones WD (2010). The Microarray Quality Control (MAQC)-II study of common practices for the development and validation of microarray-based predictive models. *Nature Biotechnology*.

[33] George SL (2008). Statistical issues in translational cancer research. *Clinical Cancer Research*.

[34] Simon R (2008). The use of genomics in clinical trial design. *Clinical Cancer Research*.

[35] Schumacher M, Hollander N, Schwarzer G, Binder H, Sauerbrei W, Crowley JJ, Hoering A (2012). Prognostic factor studies. *Handbook of Statistics in Clinical Oncology*.

[36] Fan C, Oh DS, Wessels L (2006). Concordance among gene-expression-based predictors for breast cancer. *The New England Journal of Medicine*.

[37] Molinaro AM, Simon R, Pfeiffer RM (2005). Prediction error estimation: a comparison of resampling methods. *Bioinformatics*.

[38] Jiang W, Simon R (2007). A comparison of bootstrap methods and an adjusted bootstrap approach for estimating the prediction error in microarray classification. *Statistics in Medicine*.

[39] Ambroise C, McLachlan GJ (2002). Selection bias in gene extraction on the basis of microarray gene-expression data. *Proceedings of the National Academy of Sciences of the United States of America*.

[40] Simon R, Radmacher MD, Dobbin K, McShane LM (2003). Pitfalls in the use of DNA microarray data for diagnostic and prognostic classification. *Journal of the National Cancer Institute*.

[41] Dudoit S, Fridlyand J, Speed TP (2003). Classification in microarray experiments. *Statistical Analysis of Gene Expression Microarray Data*.

[42] Varma S, Simon R (2006). Bias in error estimation when using cross-validation for model selection. *BMC Bioinformatics*.

[43] Jiang W, Varma S, Simon R (2008). Calculating confidence intervals for prediction error in microarray classification using resampling. *Statistical Applications in Genetics and Molecular Biology*.

[44] Radmacher MD, McShane LM, Simon R (2002). A paradigm for class prediction using gene expression profiles. *Journal of Computational Biology*.

[45] Dobbin KK, Simon RM (2007). Sample size planning for developing classifiers using high-dimensional DNA microarray data. *Biostatistics*.

[46] Cree IA, Kurbacher CM, Lamont A (2007). A prospective randomized controlled trial of tumour chemosensitivity assay directed chemotherapy versus physician’s choice in patients with recurrent platinum-resistant ovarian cancer. *Anti-Cancer Drugs*.

[47] Cobo M, Isla D, Massuti B (2007). Customizing cisplatin based on quantitative excision repair cross-complementing 1 mRNA expression: a phase III trial in non-small-cell lung cancer. *Journal of Clinical Oncology*.

[48] Sargent DJ, Conley BA, Allegra C, Collette L (2005). Clinical trial designs for predictive marker validation in cancer treatment trials. *Journal of Clinical Oncology*.

[49] Bogaerts J, Cardoso F, Buyse M (2006). Gene signature evaluation as a prognostic tool: challenges in the design of the MINDACT trial. *Nature Clinical Practice Oncology*.

[50] Simon R, Maitournam A (2004). Evaluating the efficiency of targeted designs for randomized clinical trials. *Clinical Cancer Research*.

[51] Maitournam A, Simon R (2005). On the efficiency of targeted clinical trials. *Statistics in Medicine*.

[52] Slamon DJ, Leyland-Jones B, Shak S (2001). Use of chemotherapy plus a monoclonal antibody against her2 for metastatic breast cancer that overexpresses HER2. *The New England Journal of Medicine*.

[53] Pusztai L, Hess KR (2004). Clinical trial design for microarray predictive marker discovery and assessment. *Annals of Oncology*.

[54] Wang SJ, O’Neill RT, Hung HMJ (2007). Approaches to evaluation of treatment effect in randomized clinical trials with genomic subset. *Pharmaceutical Statistics*.

[55] Mandrekar SJ, Sargent DJ (2009). Clinical trial designs for predictive biomarker validation: theoretical considerations and practical challenges. *Journal of Clinical Oncology*.

[56] Simon R, Wang SJ (2006). Use of genomic signatures in therapeutics development in oncology and other diseases. *The Pharmacogenomics Journal*.

[57] Song Y, Chi GYH (2007). A method for testing a prespecified subgroup in clinical trials. *Statistics in Medicine*.

[58] Jiang W, Freidlin B, Simon R (2007). Biomarker-adaptive threshold design: a procedure for evaluating treatment with possible biomarker-defined subset effect. *Journal of the National Cancer Institute*.

[59] Freidlin B, Simon R (2005). Adaptive signature design: an adaptive clinical trial design for generating and prospectively testing a gene expression signature for sensitive patients. *Clinical Cancer Research*.

[60] Freidlin B, Jiang W, Simon R (2010). The cross-validated adaptive signature design. *Clinical Cancer Research*.

[61] Matsui S, Simon R, Qu P, Shaughnessy JD, Barlogie B, Crowley J (2012). Developing and validating continuous genomic signatures in randomized clinical trials for predictive medicine. *Clinical Cancer Research*.

